# Disease outbreak data to inform decision-making: the role of disease spread models in the Lao P.D.R. African Swine Fever epidemic, 2019

**DOI:** 10.1007/s11250-025-04443-2

**Published:** 2025-05-10

**Authors:** Nina Matsumoto, Tariq Halasa, Kathrin Schemann, Syseng Khounsy, Bounlom Douangngeun, Watthana Thepagna, Phouvong Phommachanh, Jarunee Siengsanan-Lamont, James Young, Jenny-Ann Toribio, Russell Bush, Stuart D. Blacksell, Michael P. Ward

**Affiliations:** 1 Sydney School of Veterinary Science, The University of Sydney, Camden, NSW Australia; 2 Section of Animal Welfare and Disease Control, Department of Veterinary and Animal Sciences, Faculty of Health and Medical Sciences, University of Copenhagen, Copenhagen, Denmark; 3 Sydney Informatics Hub, The University of Sydney, Camperdown, NSW Australia; 4 Department of Livestock and Fisheries, Ministry of Agriculture and Forestry, National Animal Health Laboratory, Vientiane, Lao People’s Democratic Republic; 5 Faculty of Tropical Medicine, Mahidol-Oxford Tropical Medicine Research Unit, Mahidol University, Bangkok, Thailand; 6 Centre for Tropical Medicine; Global Health, Nuffield Department of Medicine, University of Oxford, Oxford, UK; 7 Lao-Oxford-Mahosot Hospital-Wellcome Trust Research Unit (LOMWRU), Mahosot Hospital, Vientiane, Lao People’s Democratic Republic

**Keywords:** African Swine Fever, Laos, Infectious disease modelling

## Abstract

Understanding the spread of African Swine Fever (ASF) between villages in the southeast-Asian, low − middle income country context is critical if this high impact disease is to be controlled by good policy and effective field activities in these resource-poor settings. Using governmental reporting data from the 2019 outbreak of ASF in Lao People’s Democratic Republic, spatial clustering techniques were used to identify clusters of outbreak villages. Then Approximate Bayesian Computation with Sequential Monte Carlo was used to estimate the transmission parameters of ASF virus between the villages within these clusters. We used a simple disease spread model to understand the impact of parameter estimation on predicted disease spread and thus decision-making. Six clusters of radius 16 to 153km were identified over the 7 month outbreak period. Within these clusters, the basic reproduction number (R_0_) ranged from 13 to 32 between-villages and whole-village infectious periods ranged from 62 to 68 days. The final model outputs were compared to the original field report data. We found that the ability of the estimated parameters to match field data was heavily reliant on how the original field surveillance data was reported. Specifically, in situations in which cases in a cluster appeared to have been reported as batches (lack of temporal specificity) our modelling approach failed to produce satisfactory outputs in terms of model fit and precision of estimates. This study demonstrates that surveillance for transboundary diseases not only has immediate benefit for disease response, but that good quality surveillance data is valuable for informing future planning for disease response via appropriately parameterised disease spread models. There is a need for ongoing quality control of surveillance and support for field veterinary services to ensure quality data that can be used to drive policy and decision-making.

## Introduction

During the 2019 African Swine Fever (ASF) outbreak in Lao People’s Democratic Republic (Lao P.D.R., henceforth Lao or Laos), the Lao Department of Livestock and Fisheries (DLF) of the Ministry for Agriculture, Forestry and Fisheries investigated and controlled over 150 outbreaks across all Provinces and territories. Stamping out measures were enacted, and control zones were placed around outbreak sites. However, the virus spread inexorably across the country over a seven-month period from the first reported outbreak in June 2019.

Resources and relevant information to determine the best strategies for ASF prevention and control in the developing country context are scarce. In developed countries, movement controls, culling, and surveillance are the key strategies for ASF control in the face of an outbreak. In the event of an outbreak in the USA, a 3 km control zone and a 10 km surveillance zone would be established around a potential outbreak (Busch et al. 2021). Chinese control zones include a 50 km buffer in areas of known wild boar activities. Regions where the disease is endemic due to vector and sylvatic hosts, such as South Africa, develop compartmentalisation strategies based on accreditation schemes (Busch et al. 2021). Understanding the unique challenges and rates of spread between sites in Laos can be used to determine whether these strategies remain effective in smallholder and transitional economy contexts.

Lao’s ASF management strategy treats entire smallholder villages the same as farms, so that control measures are implemented at the village level if an outbreak is detected (Matsumoto et al. 2021). Therefore, understanding the rate of spread and infectious period of the whole unit is critical to understanding the impact of prevention and control measures. For example, if the infectious period of a village is shortened by earlier detection and diagnosis of ASF virus (ASFV) and culling, is there a reduced rate of spread (given the high levels of free-range activity and informal trading at the village level)? There remains a lack of information about the between-village spread in ASF outbreaks in low-resource countries.

ASFV transmission parameters − such as latent and infectious periods and basic reproduction numbers (R_0_) − for individual pigs is a frequent topic of study, particularly in conventional, commercialised settings. The genotype of the ASFV in the Lao outbreaks has not yet been published; however, the neighbouring Chinese and Vietnamese outbreaks in 2018 and 2019 respectively both demonstrated p72 type II strains of the virus with genetic similarities to the Georgia 2007 strain (Tran et al. 2020; Ge et al. 2018; Le et al. 2019). In the individual pig, modelling of Chinese outbreaks demonstrated latent periods of eight to ten days and infectious periods of two to three days in the affected pigs (Li et al. 2021). These are similar infectious and latent periods to those studied in European populations, with periods ranging from two to seven and three to six days, respectively (Olesen et al. 2017; Guinat et al. 2014, 2016) and R_0_, dependent on the context of the operation, ranging from 2.67 to 16.2 (Guinat et al. 2016, 2018; Li et al. 2021). For the purposes of compartmental disease modelling, the movement of pigs between the susceptible and latent compartments per time unit is defined by a transmission term which is called the transmission rate, transmission coefficient or *β* when density dependent transmission is assumed, as is the case with the aforementioned studies. For ASFV, this estimate varies between 0.6 and 1.17 per day (Guinat et al. 2016).

In contrast to the between-pig transmission parameters, the between-unit transmission parameters of ASFV at higher-level units such as farms and villages vary considerably based on biosecurity and contact structures. The latent period would then become the period between the infection of the index case(s) and transition to the infectious period, which should be similar to that of an individual pig. The infectious period of a whole unit lasts as long as infectious individuals remain present in that unit. The R_0_ becomes the number of new units confirmed as ASF cases caused by a single unit experiencing an outbreak in a susceptible population of units. In research investigating ASFV spread between smallholder farms in Uganda, the R_0_ was estimated to be 1.63 to 3.24, and *β* to be 0.00059 to 1.90 per day, dependent on the technique for calculation (Barongo et al. 2015). In a study that estimated the time-to-clearance or time for an outbreak to either die out or infect all individuals, it was found that in Danish commercial operations of less than 1,200 pigs the period might be 50 to 120 days (Halasa et al. 2016b). These estimates represent a starting point for further estimation of transmission parameters between-villages in this study. However, for parameter estimation and therefore modelling purposes, the notable difference in management between commercial and smallholder operations require investigation.

In this study, Approximate Bayesian Methods with Sequential Monte Carlo (ABC-SMC) are utilised to estimate ASFV transmission parameters between outbreak villages in Laos. Since traditional compartmental disease modelling assumes homogenous mixing, spatiotemporal cluster analysis was utilised to identify outbreak clusters amongst villages of Laos during the 2019 outbreak. A simple disease spread model was used as a tool to understand what might be the impact of data quality on predicted disease spread and therefore the information provided to decision-makers. This is invaluable for ASF management in the Lao context, and these findings can be applied more broadly to understand epizootic control in the Southeast Asian region.

## Materials and methods

### Data collection

#### Outbreak data

The reference population was all villages confirmed as ASFV positive in 2019 using rt-PCR at the Lao National Animal Health Laboratory (NAHL). The outbreak study period was between 01 June 2019 and 01 January 2020. This information was stored in the Pathogen Asset Control System (PACS) provided by the US-DTRA funded CAMN3 and CAMN4 programmes. With the exception of the ten villages in Savannakhet and Oudomxay investigated by Matsumoto et al. 2021, 2023), none of the villages had the dates of pig mortalities commencing at the within-village level. Instead, each village had a reporting date linked to the sample and outbreak report submitted by the local DLF officers and a testing date linked to the date on which that sample was confirmed ASFV positive. As a result, the reporting date was used as the “mortality date” for modelling purposes. For outbreaks in Oudomxay Province and Savannakhet Province, the dates of the first report were derived from data provided by the Provincial DLF staff in previous outbreak investigations of Lao smallholder villages (Matsumoto et al. 2021, 2023).

#### Location data

Lao’s administrative divisional structure consists of Provinces, Districts and Ban (villages). Each outbreak location was identified in the PACS data by Province, District and Village but not by latitude and longitude. The latitude and longitude coordinates were derived from the 2011 Lao Agricultural Census and the DIVA-GIS Gazetteer. From 152 villages included in the original dataset, 147 were identifiable using the available data sources. When the village was not identifiable, a random point within the district was chosen based on the district's northern, southern, eastern, and western edges (*n* = 3) using http://www.geomidpoint.com/random/. If the above strategy could not be applied, the village was not included in the spatial analysis (*n* = 2). The final dataset contained 150 outbreak villages representing all Provinces in Laos.

#### Spatial cluster identification

In the ABC-SMC technique, a simple compartmental disease model was used to validate the simulated parameter data. Due to the assumption of homogenous mixing in a modelled population, the outbreak data were analysed for spatiotemporal clusters, and modelling was then performed on the identified clusters individually.

Retrospective space–time permutation (STP) analysis was performed in SaTScan v9.6 (Kulldorff 2010) to identify spatial clusters of ASFV cases in the outbreak data. STP can be visualised by drawing a circle on the map and calculating how many cases are inside and outside the circle. At the same time, this circle extends upwards on the z-axis to represent multiple days. If there are more cases inside the scanning window than expected for that area and that period of the outbreak, it is considered a cluster. Scanning windows of various diameters (area) and heights (time) are then permuted to identify clusters representing different sizes (square kilometres) and lengths of time (days), respectively.

STP analyses use only case data; this is a preferable approach in the Lao ASF situation where the likelihood of non-reporting bias is high (Miller et al. 2018). Where more reliable case and population or control data become available, Poisson or Bernoulli analyses could be performed to provide additional insight into clustering (Durr and Gatrell 2004).

The analysis used the individual day as the time unit. The maximum spatial cluster size was 50% of the population at risk (case villages), and the temporal window was set to a maximum length of 60 days. Results from significant clusters (*P* < 0.05) were subsequently used for model building.

### Parameter estimation

#### Adjustments to the between-pig disease model for the between-village context

Within the ABC-SMC model, a simple susceptible (S) – latent (E) – infectious (I) – recovered (R) or SEIR model was used to model the transmission of ASFV between villages within the significant clusters identified in STP analysis. This utilised ordinary differential equations for movement between states (Eqs. 1–4).

1 $$\frac{dS}{dt}= -\beta SI,$$

2 $$\frac{dE}{dt}= \beta SI-\sigma E,$$

3 $$\frac{dI}{dt}= \sigma E- \gamma I,$$

4 $$\frac{dR}{dt}= \gamma I,$$

where 1/σ is the mean duration of the latent period (µ_e_), and 1/γ is the mean duration of the infectious period (µ_i_) (Keeling and Rohani 2011). These probability distributions were assumed to follow gamma distributions using the parameters mean and shape. In this study the gamma distributions utilised means µ_e_ and µ_i_ and shapes k_e_ and k_i_ for latent and infectious respectively (Guinat et al. 2018; Keeling and Rohani 2011). Here, only k_e_ was estimated since there were no appropriate existing infectious period data provided information about the k_i_. *β* is the disease transmission coefficient, which depends on the effective contact rate between villages and the probability of disease transmission given contact.

The formula for R_0_ in an SEIR model is derived using *β* and the mean infectious period (1/γ) (Eq. 5) (Keeling and Rohani 2011, 97).

5 $${R}_{0}=\upbeta \frac{1}{\upgamma }$$

Due to the previously mentioned constraints of the available data in identifying controls, and the risk of non-reporting, it was decided that only the case villages within each identified cluster should be modelled.

#### ABC-SMC summary

The Approximate Bayesian Computation with Sequential Monte Carlo (ABC-SMC) technique is described in Guinat et al. (2018). The purpose of re-using this technique with different priors was to test the outputs and adaptability of the method when scaling to larger epidemiologic units with fewer data points.

In the ABC-SMC, a simple disease simulation model was run using a particle of transmission parameters. The parameters forming the particles in the first generation were randomly selected from informed priors, and the outputs (predictions) were compared to the field (observed) data. If the simulation performed within a pre-determined threshold of similarity to the field data, the particle was carried through to the next generation. Within each generation, 10,000 parameters were collected and slightly perturbed based on their marginal distribution at the end of the previous generation to then be passed to the subsequent generation. The posterior distributions after 25 generations are reported as the final distribution.

In this study, 25 generations were completed. The sum of squares (SS) between the cumulative daily number of ASFV positive villages in the reporting data and the disease simulation using the particle of transmission parameters was the measure of similarity (Eq. 6). The initial tolerance (ε) was an SS of 75, with the 75 th percentile of the SS being the cut off for each subsequent generation. The model was considered to have stabilised when ε stabilised. The parameters estimated are shown in Table 1.

**Table 1:** Priors used in between-village ABC-SMC transmission parameter estimation for ASFV in Lao PDR, 2019

Parameter	Symbol	Informative prior (mean, shape)	Sources
Between-village transmission rate	$\beta$	1.22, 0.43	Barongo et al. (2015)
Shape parameter—latent period	k_E_	19.39, 5	Guinat et al. (2018); Guinat Gubbins et al. (2016); Hu et al. (2017); Korennoy et al. (2017)
Mean latent period	µ_E_	7, 17	
Mean infectious period (Village)	µ_I_	100, 10	Halasa et al. (2016b)

6 $$sum\, of\, squares= \sum\limits_{{t= t}_{1}}^{{t}_{2}}{({mortalities}_{simulated(t)}-{mortalities}_{field(t)})}^{2}$$

#### Priors and assumptions

Bayesian methodologies allow the observed data to be compared to existing data and inferences drawn based on these observations. For this reason, prior data used in Bayesian modelling should be as unbiased and representative of the context as possible, keeping in mind that there is a lack of relevant data about transmission parameters at the smallholder, between-village level.

The $\beta$ prior was developed by converting all the described estimates for ASFV outbreaks between smallholder farms in Uganda (0.0059, 1.77 and 1.9) into a gamma distribution using the egamma() function in the EnvStats package of RStudio (Barongo et al. 2015). Due to the uncertainty of the background data, a wide uniform prior of 0.0001 to 3.0 was also investigated.

The latent period shape (k_E_) and mean (1/σ or ${\mu }_{E}$) were derived from the work of (Guinat et al. 2018), based on the assumption that the latent period of a village should be the same as the latent period of the first pig or pigs that were infected in that village.

## Results

### Descriptive statistics

Between June and December 2019, 152 outbreaks were reported to the DLF across all the Provinces. The earliest outbreak report occurred in Salavane Province (16 June 2019) and the last report for the period occurred in Vientiane Capitol (23 December 2019). The period over which outbreaks were reported ranged from 0 to 173 days. In the Provinces where the period = 0, all reports were submitted on the same day (Table 2).

**Table 2:** Outbreak descriptive data from the DLF by Province in Lao PDR, 2019

Province	Cases	First Outbreak (2019)	Last Outbreak (2019)	Period (days)
Attapeu	8	8 August	27 August	19
Bokeo	20	10 September	27 September	17
Bolikhamxay	15	2 August	1 October	60
Champasack	1	11 September	11 September	0
Houaphan	17	15 July	25 October	102
Khammouane	1	18 November	18 November	0
Luang Namtha	9	4 August	8 December	126
Oudomxay	31	18 July	18 November	123
Phongsaly	10	16 July	5 September	51
Salavane	7	16 June	8 July	22
Savannakhet	5	3 July	18 September	77
Vientiane Capital	8	3 July	23 December	173
Vientiane	2	1 August	30 August	29
Xaysomboun	5	13 July	10 October	89
Xekong	6	1 August	18 September	48
Xieng Khouang	5	4 August	15 September	42

### Cluster identification

**Fig. 1:**
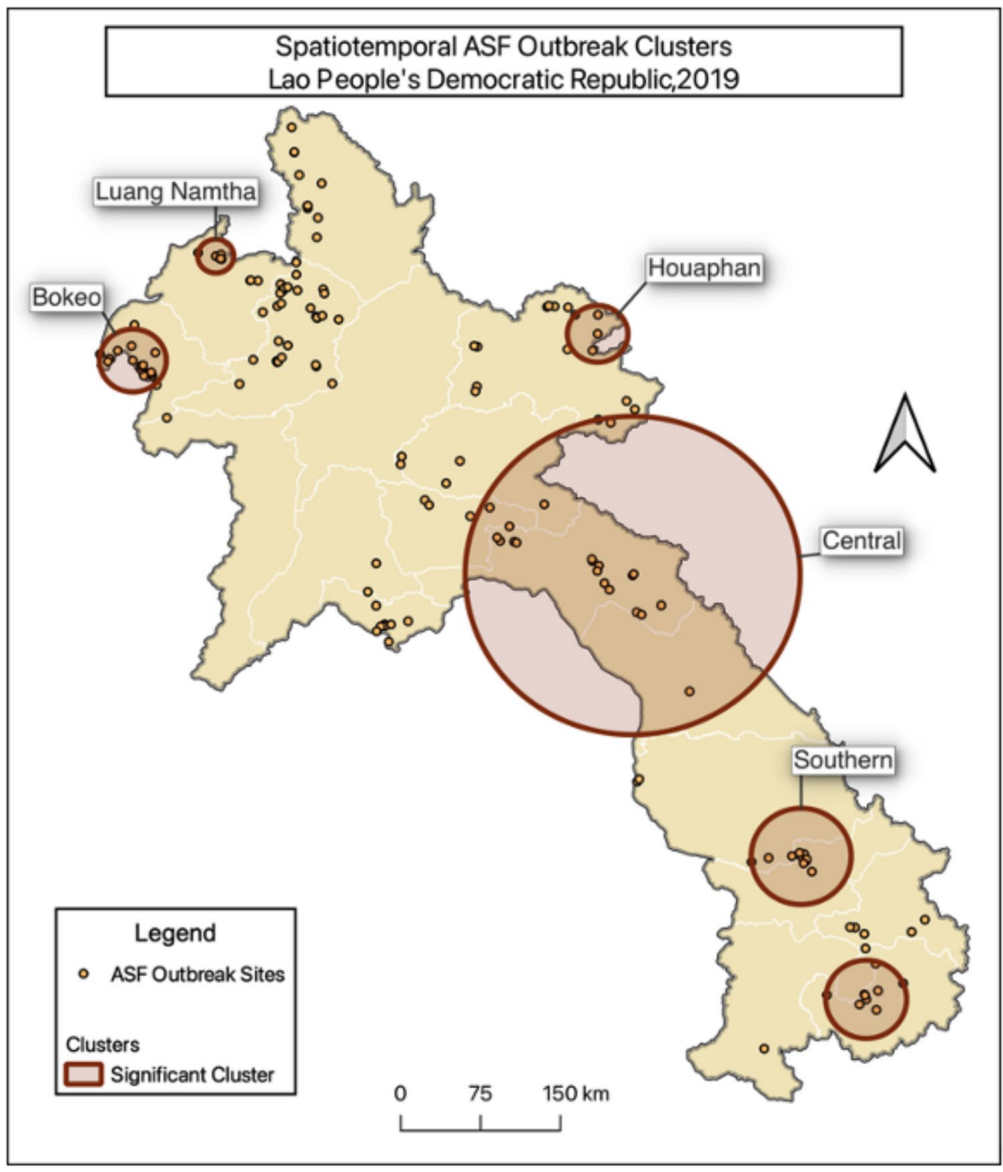
Map of ASF-affected village clusters identified using spatiotemporal permutation analysis in Lao PDR, 2019

STP analysis identified six spatiotemporal clusters in the DLF ASFV reporting data from June 2019-January 2020 (Fig. 1). Listed chronologically in Table 3, these clusters contained 4 to 14 case villages during the identified cluster periods, with radii ranging from 16 to 153 km. If the cases involved multiple Provinces, they were named based on their location. The Central cluster included Bolikhamxay, Khammouane, Houaphan and Xaysomboun Provinces. The Southern cluster was made up of Savannakhet and Salavane Provinces.

**Table 3:** ASF clusters identified using spatiotemporal permutation analysis in Lao PDR, 2019

Cluster	Dates (2019)	Villages in cluster	Radius (km)
Southern	16 June – 08 July	10	46
Houaphan	22 July – 22 July	4	27
Central	23 July – 30 July	14	153
Attapeu	13 August – 27 August	7	38
Luang Namtha	05 September – 05 September	4	16
Bokeo	13 September– 14 September	14	30

### Parameter estimation outputs

The mean latent period was 5.82 to 5.96 days across the clusters, representing the latent period of the index case or cases within the village. The infectious period for the whole village was estimated to be 61.53 to 67.70 days, representing how long infectious pigs were within the village and able to transmit ASFV either directly or indirectly from village to village within the clusters. The mean *β* for transmission within a cluster was 0.19 to 0.51. The R_0_ ranged from 13.06 to 31.63 across the clusters (Table 4). The shape parameter for k_E_ was 15.97 to 16.64.

**Table 4:** Estimated transmission parameters between villages in ASFV clusters identified in the Lao 2019 outbreak using beta priors

Cluster	R_0_	β	µ_E_	µ_I_	ε
Southern	31.63 (31.57–31.7)	0.51 (0.41–0.61)	5.82 (4.61–7.11)	61.53 (56.62–63.63)	8.91
Houaphan^1, 2^	23.22 (23.18–23.26)	0.37 (0.31–0.43)	5.88 (4.72–7.13)	63.76 (58.36–66.04)	12.66
Central	19.18 (19.15–19.21)	0.3 (0.26–0.35)	5.87 (4.65–7.1)	65.83 (60.72–68.11)	7.21
Attapeu^1^	16.44 (16.41–16.46)	0.25 (0.22–0.28)	5.92 (4.73–7.12)	66.72 (60.39–69.5)	9.46
Luang Namtha^1, 2^	16.43 (16.41–16.46)	0.25 (0.22–0.28)	5.95 (4.72–7.21)	66.64 (59.99–69.34)	7.36
Bokeo^2^	13.06 (13.043–13.08)	0.19 (0.17–0.22)	5.96 (4.77–7.2)	67.7 (60.43–70.77)	12.41

R_0_ – basic reproductive number, β – between-village transmission rate,${\mu }_{e}$- mean latent period (village),${\mu }_{i}$- mean infectious period (village), ε – mean epsilon for final round; 1 – less than 10 villages in cluster, 2 – reports occurred over 1–2 days; all results presented are the mean of the posterior distribution with 95% CI

### Model performance

#### Final SEIR outputs

For all models, the parameters used as priors resulted in epidemics occurring in each simulated cluster. Analysis of the outputs demonstrated that for all parameters the median estimate stabilised, and the marginal distribution decreased appropriately over the course of 25 generations in all clusters.

**Fig. 2:**
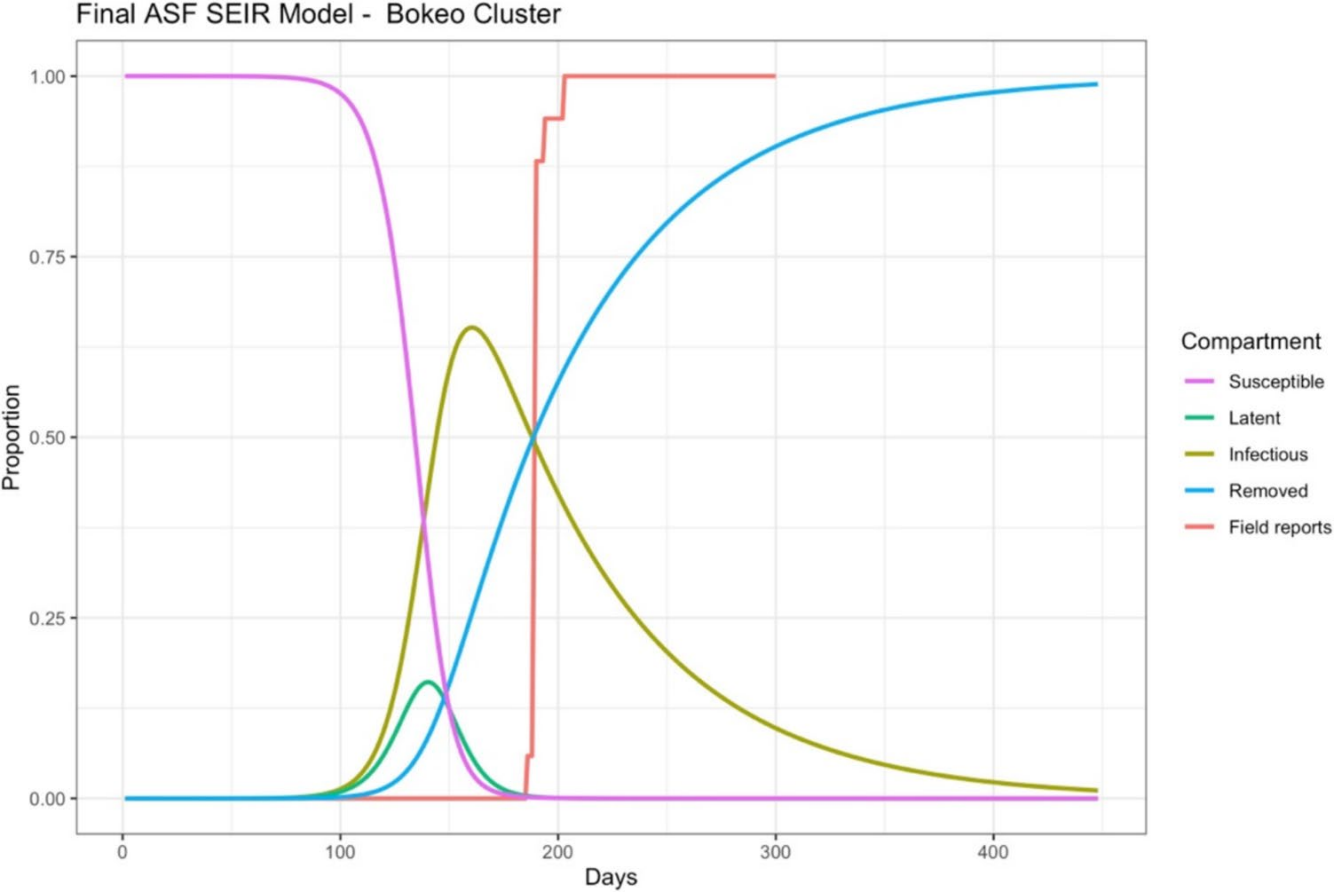
Simulated outbreak using parameter estimates for the Bokeo ASF outbreak cluster in Lao PDR (2019)

**Fig. 3:**
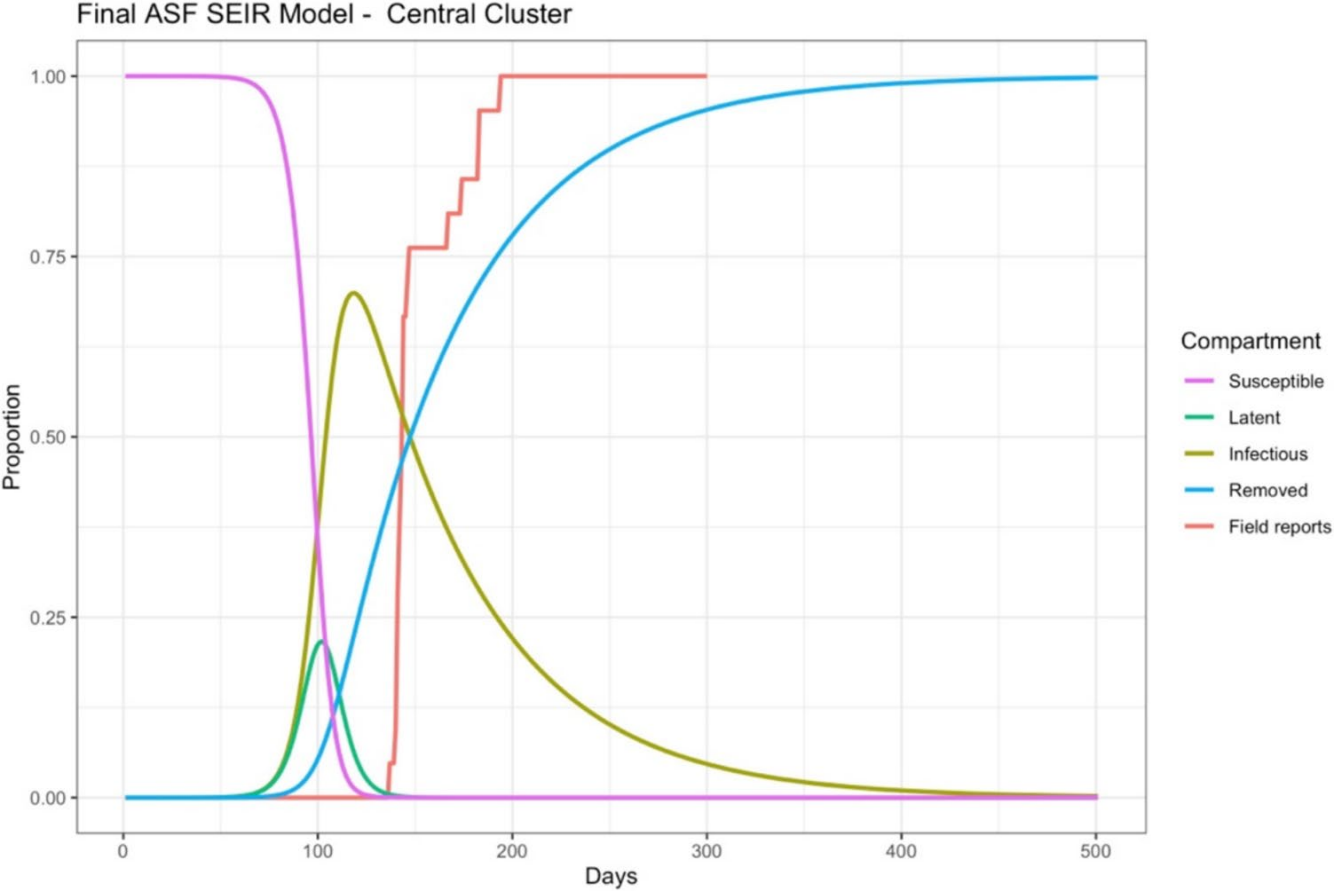
Simulated outbreak using parameter estimates for the Central ASF outbreak cluster in Lao PDR (2019)

**Fig. 4:**
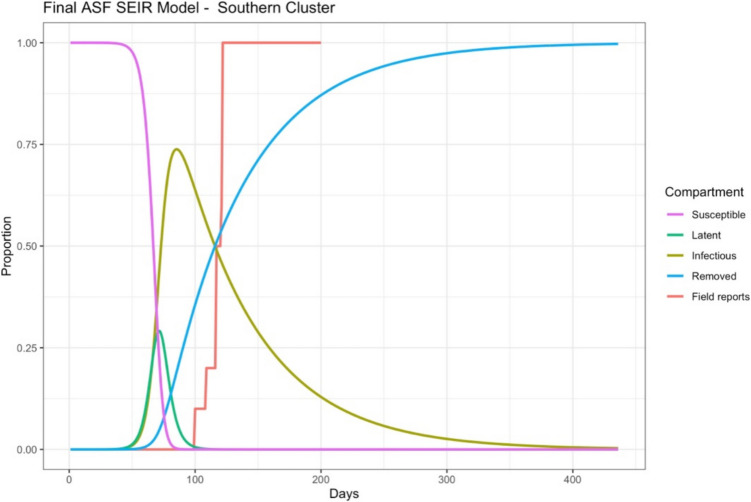
Simulated outbreak using parameter estimates for the Southern ASF outbreak cluster in Lao PDR (2019)

**Fig. 5:**
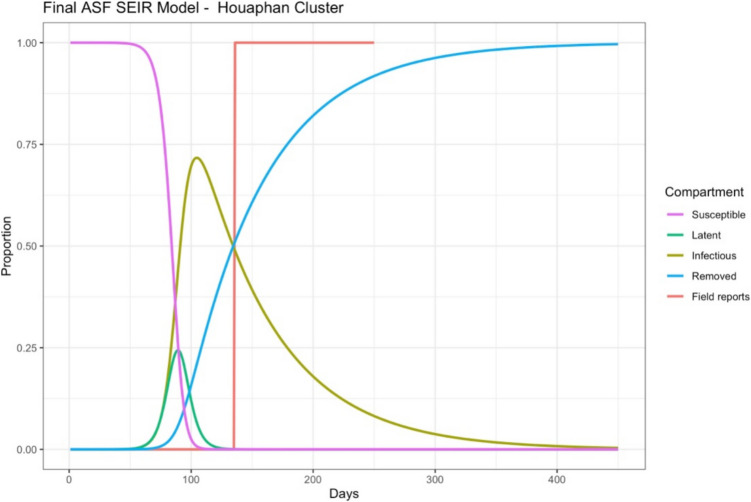
Simulated outbreak using parameter estimates for the Houaphan ASF outbreak cluster in Lao PDR (2019)

**Fig. 6:**
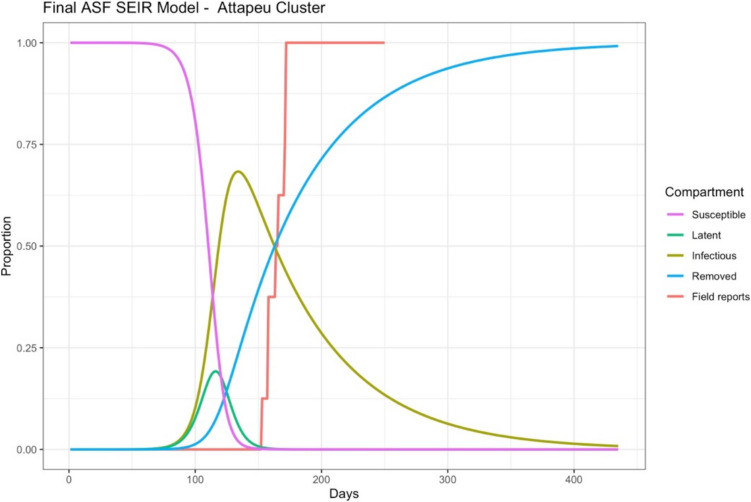
Simulated outbreak using parameter estimates for the Attapeu ASF outbreak cluster in Lao PDR (2019)

**Fig. 7:**
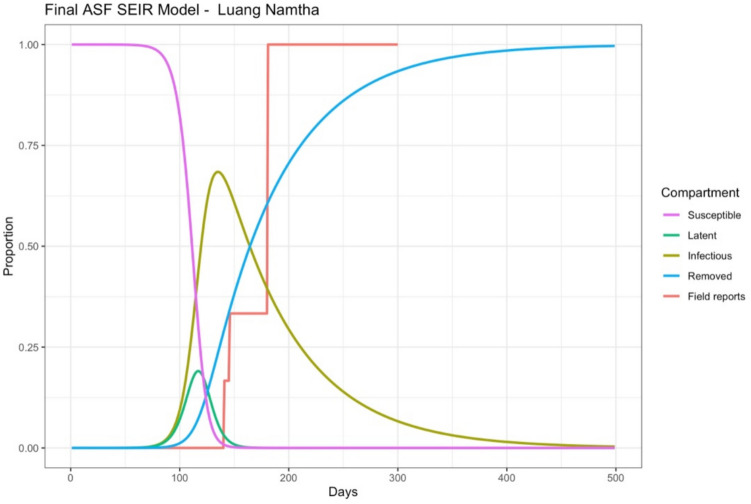
Simulated outbreak using parameter estimates for the Luang Namtha ASF outbreak cluster in Lao PDR (2019)

The final models utilising the estimated transmission parameters demonstrate that sites with less outbreaks (less than ten villages) or where all outbreaks were reported in one to two days bisected – but struggled to match – the DLF data (Fig. 2). Whilst clusters with multiple reports over several days demonstrated a closer match between the modelled removals (blue lines) and the DLF field reporting data (red lines) (Fig. 3). Additional model outputs have been supplied in Appendix 1 (Figs. 4, 5, 6 and 7).

#### Epsilon

All analysed clusters showed stabilization of *ε* at 25 generations, with the mean *ε* in the final generation performing best in the Central cluster (7.21) and worst in the Houaphan and Bokeo clusters (12.66 and 12.41 respectively) (Table 4).

## Discussion

We provide initial estimates for infectious periods between smallholder villages and R_0_ values, which can assist our understanding of the possible scope of an ASF outbreak in a smallholder setting. This research also highlights the limitations and challenges of such approaches in data-scarce environments where local resources require investigators to utilise imperfect or incomplete data. Ultimately, this methodology and its limitations are intended for utilization in the modelling of ASFV spread and control in outbreak scenarios. However, key flaws in the input surveillance data suggest that future studies should investigate the performance of the output parameter estimates in a stochastic national model to assist in fine-tuning the estimates.

The quality of the input reporting and surveillance data had a direct impact on the final outputs of the models and therefore the generalisability of the results and the decisions that are made. The outbreak reports utilised in this study were influenced by factors such as financial and staff resource availability, distances from the local Provincial DLF staff and the NAHL. This lack of systematic reporting and support is reflected in the final outputs i.e. some Provincial DLF staff chose to report multiple sites on the same day to increase the financial efficiency of their operations. Upon inspection of the final SEIR model outputs, it is apparent that models based on data with at least ten case villages and reported over a period of seven or more days performed best in parameter estimation. Three of the identified clusters occurred over two days or less. This may not represent a true cluster of disease incidence but rather a cluster of disease reporting activities. In Savannakhet Province, the local DLF staff reported attending all ASF-affected sites over the course of a few days to collect samples for official ASFV diagnosis. However, outbreak investigation from the same sites demonstrated that the mortalities occurred over a much longer period, prior to the official report date (Matsumoto et al. 2021). The impact of disease reporting systems on the data quality is of note for future applications of the technique, or when applying exact Bayesian methods in the same context. This is particularly relevant to understanding whether or not the reported case villages truly represented *all* infected villages, or only the villages that chose to report and were investigated. In the current study the focus was on the initial spread of a novel disease in Laos, ASF. Therefore, lack of reporting might be assumed to be negligible. Ongoing improvement of surveillance and reporting mechanisms should be pursued to aid data quality improvement and outbreak response times. This could be achieved by utilising techniques such as targeted active surveillance, syndromic surveillance and streamlining disease reporting systems as has been trialled in Laos (Gee et al. 2024).

Despite variable data quality, the estimates for latent and infectious periods (5.82 to 5.95 and 61.53 to 67.70 days respectively) were consistent across all modelled clusters. The between-village R_0_ estimates of 13.06 to 31.63 are much higher than those of smallholder farms in Uganda and commercial farms in Denmark and Russia, however the values for β are consistent with previous studies (Barongo et al. 2015; Halasa et al. 2016b; Gulenkin et al. 2011). The estimate represents the number of contacts during the infectious period and the probability of transmission during a contact event, further highlighting the need for more detailed data on between-village contact structures and the transmission behaviours on contact. R_0_ is also dependent on the behaviours between groups; for example, in regions where travel between villages is more difficult due to poor road conditions R_0_ estimates between villages might be lower than in regions that are more interconnected. Causes for increased rates of contact between villages should be investigated for future targeted disease prevention and control efforts.

The estimates for the latent period of a village are consistent with the literature on ASFV in pigs: latent periods estimated for pigs in experimental studies include four to five days for the Malta 1978 and Netherlands 1986 strains (de Carvalho Ferreira et al. 2013) and 3.6 to 5.4 days for Caucasian and Georgia 2007 strains (Claire Guinat et al. 2014; Pietschmann et al. 2015). This demonstrates that despite variable input data quality, the estimation model was able to produce realistic outputs.

Between-village ASFV transmission can occur either through direct contact (such as an emergency sale of an unwell herd) or indirect contact (such as fomite transmission on vehicles or personnel). In this study the most relevant data available was estimation of the time to ASFV clearance – or time until every animal is infected with ASFV, or the disease has died out, with no interventions. The time to clearance of ASFV from a herd was estimated in small (less than 300 pigs) and medium (301 to 1200 pigs) commercial pig operations in Denmark to range between 50 and 120 days depending on unit size and the transmission rate in the outbreak (Halasa et al. 2016a). Given the previous work of the authors investigating ASF-affected households in smallholder villages, the herd size of the village appears to mirror that of either a small or medium herd depending on the region, and mortalities occurred over periods of 22 to 103 days (Matsumoto et al. 2021, 2023). Understanding the infectious period of a village provides insight into the spread of the disease within the village, as well as providing a valuable timeframe over which trace-back and trace-forward activities should take place.

Future work investigating possible mechanisms of spread such as familial visits, weddings, and festivals, sharing of contagious equipment, informal trading activities or wild boar movements would inform the pathways by which diseased pigs or pig products were able to spread from village to village. The ASF epidemic progressed throughout Asia in a reasonably continuous pattern from north the south and from east to west (Mighell and Ward 2021). During this period there were no reports from Thailand (so no edge effects) and earlier reports from Vietnam (Mighell and Ward 2021). At the continental scale there was only the one cluster identified in southeast Asia that included Laos and Vietnam (and southern China) (Mighell and Ward 2021). The cluster data presented here represent only the outbreaks recorded within Laos, however cases were also occurring in Vietnam and Cambodia simultaneously. This likely created an edge effect, where clusters may have occurred nearby to national borders, but the STP model was not able to identify them.

The estimated transmission parameters support the idea that ASFV is of moderate contagiousness, consistent with the findings of many other recent studies comparing it to diseases such as FMD or CSF, which are highly contagious. Despite the slower rate of spread, the mortality rates for ASFV infection are devastating, ranging from 54–99% on initial presentation of veterinary services in Oudomxay (Matsumoto et al. 2023). The aim of understanding these transmission parameters is to utilise them in modelling the control of the disease. For example, if movement control measures could have been implemented earlier using a point-of-care test at the time of investigation and prior to laboratory confirmation – then perhaps the effective contact rate between villages would have been reduced and therefore the rate of spread slowed. Slower rate of spread allows more time for veterinary services to implement prevention and control measures, to mitigate the significant financial and food security impacts reported due to ASF in South-East Asian countries (Smith et al. 2019; Berends et al. 2021; Hui et al. 2023). This is highly relevant in a nation like Laos, where 30% of all farmers keep pigs (MAF 2010). It is therefore paramount that these parameters be estimated accurately, using alternate techniques or data collection approaches in future.

The stamping out and movement control measures for ASF in Laos should be assessed in more detail, as the radii of the identified clusters were far larger than the 3 and 10 km regions commonly utilised in Europe. Furthermore, the presence of large numbers of potential disease reservoirs and transmission pathways, such as wild boar, informal trading and free-range pigs may reduce the efficacy of a stamping out strategy on its own. By combining these findings with ongoing surveillance data, continued outbreak investigation and detailed disease and financial modelling, the control of ASF can be mitigated in Laos to protect the future of smallholder livelihoods.

## Conclusions

Utilising ABC-SMC methods to estimate transmission parameters for between-village spread of ASFV requires high quality input data to provide useful outputs. Based on reported surveillance data from the Lao 2019 outbreak and the disease clusters identified, villages remain infectious for 62 to 68 days, with R_0_ estimates ranging from 13 to 32. The ability of the models to match the field surveillance data were highly dependent on manner of data reporting and specifically representation of the evolution of each epidemic. This study highlights the need to support field veterinary services in these areas to not only respond to disease outbreaks but to investigate and report high quality information to inform future disease control and decision-making.

## Data Availability

Data can be made available upon reasonable request.
